# Oral cancer induced TRPV1 sensitization is mediated by PAR_2_ signaling in primary afferent neurons innervating the cancer microenvironment

**DOI:** 10.1038/s41598-022-08005-6

**Published:** 2022-03-08

**Authors:** Nicole N. Scheff, Ian M. Wall, Sam Nicholson, Hannah Williams, Elyssa Chen, Nguyen H. Tu, John C. Dolan, Cheng Z. Liu, Malvin N. Janal, Nigel W. Bunnett, Brian L. Schmidt

**Affiliations:** 1 Department of Neurobiology and Hillman Cancer Research Center, University of Pittsburgh, Pittsburgh, USA; 2 Department of Oral and Maxillofacial Surgery, Bluestone Center for Clinical Research, New York University (NYU) College of Dentistry, New York, USA; 3 Pathology Department, NYU Langone Health, New York, USA; 4 Department of Epidemiology and Health Promotion, NYU College of Dentistry, New York, USA; 5 Department of Molecular Pathobiology, NYU College of Dentistry, New York, USA; 6 Department of Neuroscience and Physiology, Neuroscience Institute, NYU Langone Health Neuroscience Institute, NYU Langone Health, New York, USA

**Keywords:** Head and neck cancer, Pain

## Abstract

Oral cancer patients report sensitivity to spicy foods and liquids. The mechanism responsible for chemosensitivity induced by oral cancer is not known. We simulate oral cancer-induced chemosensitivity in a xenograft oral cancer mouse model using two-bottle choice drinking and conditioned place aversion assays. An anatomic basis of chemosensitivity is shown in increased expression of TRPV1 in anatomically relevant trigeminal ganglion (TG) neurons in both the xenograft and a carcinogen (4-nitroquinoline 1-oxide)-induced oral cancer mouse models. The percent of retrograde labeled TG neurons that respond to TRPV1 agonist, capsaicin, is increased along with the magnitude of response as measured by calcium influx, in neurons from the cancer models. To address the possible mechanism of TRPV1 sensitivity in tongue afferents, we study the role of PAR_2_, which can sensitize the TRPV1 channel. We show co-expression of TRPV1 and PAR_2_ on tongue afferents and using a conditioned place aversion assay, demonstrate that PAR_2_ mediates oral cancer-induced, TRPV1-evoked sensitivity in an oral cancer mouse model. The findings provide insight into oral cancer-mediated chemosensitivity.

## Introduction

Oral cancer patients endure severe pain while undertaking routine oral functions that mechanically agitate soft tissue in the oral cavity such as eating and talking^1,2^. On the other hand, these same patients report low levels of spontaneous pain. Using xenograft and carcinogen-induced oral cancer mouse models and orofacial assays, we have modeled and studied the functional/mechanical pain observed in oral cancer patients^3^. Anecdotal clinical evidence reveals that some oral cancer patients also suffer from chemosensitivity, especially to spicy foods and liquids. We have not studied this symptom to date. Substances within spicy food activate the transient receptor vanilloid channel 1 (TRPV1). While afferent neurons throughout the different heterosegmental regions express TRPV1, the oral cavity is an ideal site to study TRPV1 sensitization in the setting of cancer. Exposure of the soft tissue of the oral cavity to capsaicin is consistent with the eating habits of numerous cultures. Accordingly, application of capsaicin to the tongue in a rodent oral cancer model mirrors the experience of oral cancer patients who eat or drink spicy ingredients. A model that utilizes subcutaneous injection of capsaicin does not accurately recapitulate the topical exposure to spicy substances that patients experience.

Little is known regarding cancer-induced chemosensitivity. However, various cancer pain models involving several species reveal that TRPV1 is upregulated on sensory neurons that innervate oral cancers. Moreover, TRPV1 mediates oral cancer pain^4–8^. For example, in an oral cancer pain model generated by inoculating squamous cell carcinoma into the gingiva of a rat, TRPV1 is overexpressed in the associated trigeminal ganglion (TG); mechanical allodynia and thermal hyperalgesia are observed in this model^9^. Agonists in the cancer microenvironment, including an elevated proton concentration, could activate TRPV1^10–13^. Rhabdomyosarcoma and osteosarcoma secrete lipophilic substances that activate TRPV1 on TG neurons^14^. In one of the most promising studies to date, selective ablation of TRPV1 fibers with intrathecal resiniferatoxin reversed cancer pain and restored function in a canine bone cancer model^15^.

TRPV1 sensitization, rather than activation, is consistent with oral cancer pain during routine oral function, but not with spontaneous pain. TRPV1 sensitization occurs through phosphorylation of intracellular residues on the ion channel, a process that is modality specific^16^. Protease-activated receptor-2 (PAR_2_), which has a prominent role in oral cancer pain^3,17^, activates protein kinase C and A and leads to ion channel phosphorylation and sensitization of TRPV1^18^. The aim of our study was to determine whether oral cancer leads to TRPV1-mediated chemosensitivity, and whether the mechanism involves PAR_2_, which is unknown in the setting of oral cancer. To study upregulation of TRPV1 on anatomically-relevant sensory neurons innervating the tongue, which we identified with the retrograde tracer DiI, in the setting of oral cancer, we used two oral cancer mouse models, the xenograft model with athymic nude mice and a carcinogen-induced oral cancer mouse model with wildtype C57BL/6 mice. We used PAR_2_ knockout mice and conditioned place aversion (CPA) to assess the role of PAR_2_ in oral cancer-induced TRPV1 sensitization. A better understanding of TRPV1 sensitization in the setting of cancer could provide insight into how TRPV1-mediated cancer nociception could be alleviated or preempted.

## Results

### Tongue primary afferents express TRPV1 protein that mediate aversion to capsaicin in the drinking water

**Figure 1:**
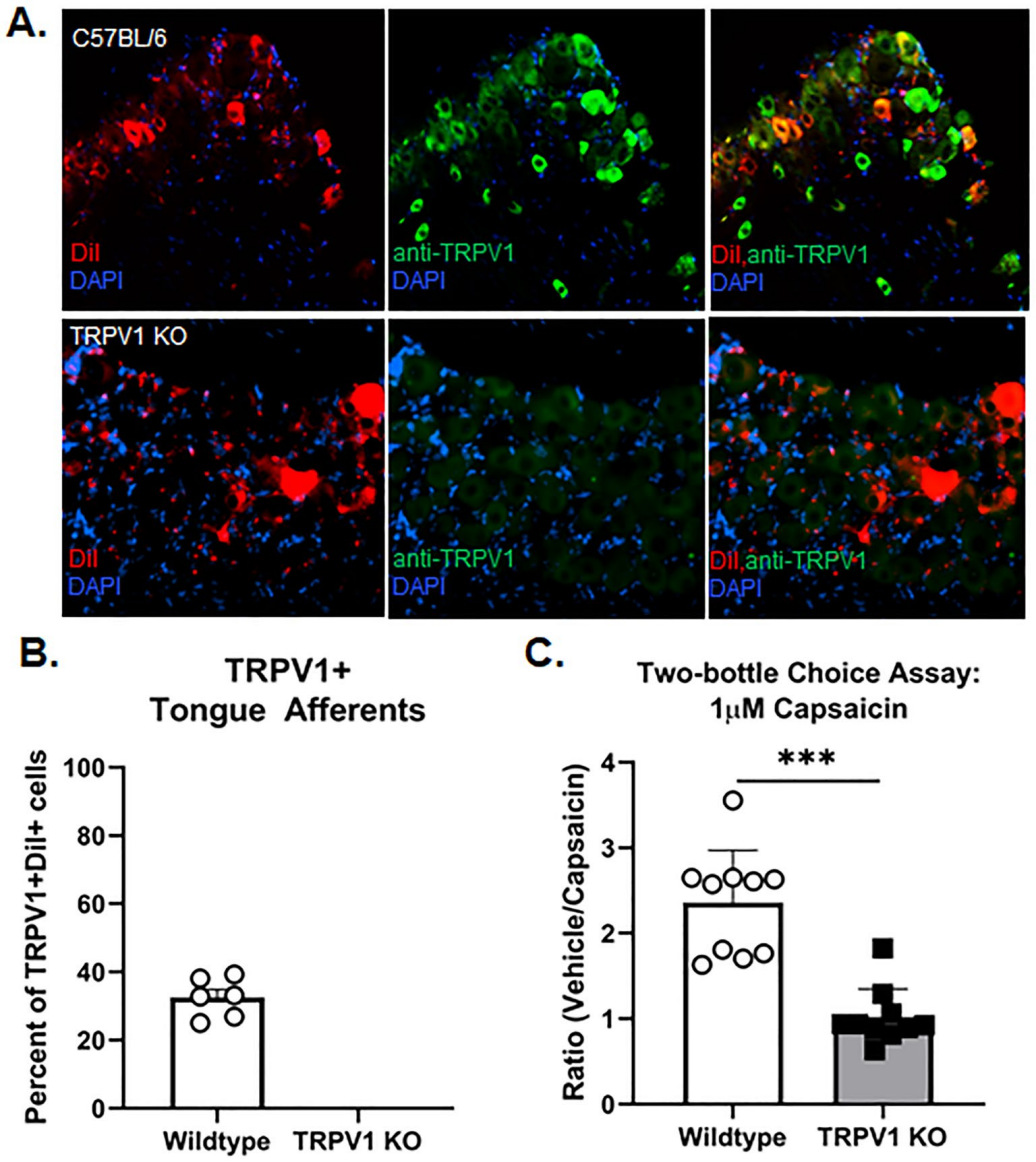
Tongue primary afferents express TRPV1 protein that mediates aversion to capsaicin in the drinking water. (**A**) Representative images of TRPV1-like immunoreactivity in TG sections from a retrograde labeled (DiI) female naïve C57BL/6 or female TRPV1 knockout (KO) mouse. (**B**) The pooled data indicate the percent of DiI + (red) neurons that were co-labeled with TRPV1 (green) from male and female C57BL/6 mice compared to TRPV1 KO mice (n = 3/sex). (**C**) There was a genotypic difference in TRPV1-mediated aversion to 1 µM capsaicin; wildtype C57BL/6 male and female mice (n = 5/sex) demonstrate an aversion to capsaicin in the drinking water compared to TRPV1 KO mice which demonstrated no preference. ***p < 0.001.

A significant portion of sensory nerve innervation to the tongue arises from neurons in the V3 region of the TG. We determined the percentage of retrograde labeled tongue afferents that express TRPV1 in order to gauge the functional impact of TRPV1 activation on aversion behavior. Using a retrograde tracer, the subpopulation of TRPV1-positive TG neurons that innervate the tongue was 32.95 ± 3.5% (n = 3/sex/group, Fig. 1A,B). We found no positive staining in the TRPV1 KO mouse (Fig. 1A,B). A two-bottle drinking aversion test was used to measure TRPV1 mediated oral sensitivity. Two bottles, one that contained water with vehicle (0.1% ethanol) and the other containing water with 1 µM capsaicin, were available for 5 days to single sex socially housed male and female mice (n = 3/cage/sex). TRPV1 KO mice were used as a comparison. Total water consumption (mL) was the same across sex and genotype (sex by genotype: F(1,16) = 0.013, p = 0.909; independent samples t-test with pooled sexes: t(16) = 0.152 p = 0.881). However, capsaicin water was avoided in wildtype mice, where both male and female C57BL/6 mice drank less than half the volume of water containing capsaicin compared to TRPV1 KO mice (Fig. 1C, t(16) = 7.023, p < 0.001).

### TRPV1-mediated aversion to capsaicin during oral cancer progression

**Figure 2:**
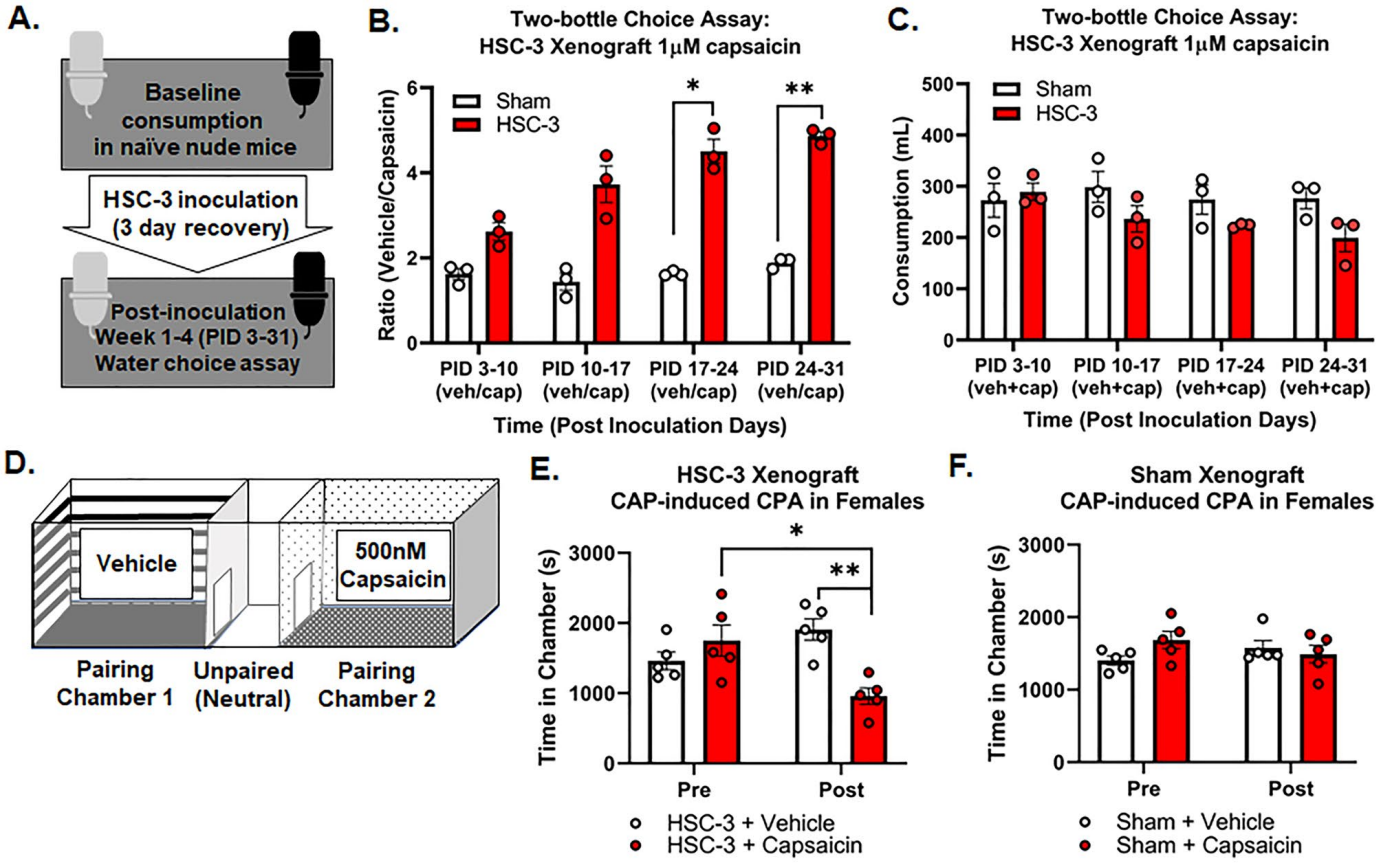
TRPV1-mediated aversion to capsaicin during oral cancer progression (**A**) Timeline for the two-bottle drinking assay using the oral cancer xenograft mouse model. Baseline water consumption using vehicle water (0.01% EtOH) was recorded prior to cancer cell injection into the tongue. (**B**) Tumor-bearing female athymic nude mice demonstrate greater aversion to 1 µM capsaicin in the drinking water compared to sham at 3 and 4 weeks post inoculation. We measured water consumed by cage; each cage housed 3 mice; each dot represents a cage of three female mice. Time x treatment, p = 0.0013. *p < 0.05, **p < 0.01 (**C**) There was a similar reduction in total consumption with cancer progression in tumor-bearing mice regardless of time and treatment. p > 0.05. (**D**) Standard CPA box in which mice were conditioned with either saline or 500 nM capsaicin oral swabbing on post inoculation days 22–24. Place aversion was measured on day 25 and CPA was assessed as the difference in time spent in the capsaicin-paired chamber compared to saline-paired chamber. (**E,F**) Tumor-bearing female mice (white and red bars) spent less time in the capsaicin-paired chamber compared to baseline. Time x treatment p = 0.0056, *p < 0.05, **p < 0.01. Capsaicin treatment had no effect in sham female mice. Time x treatment p = 0.1207.

TRPV1 activation is thought to be involved in cancer pain^19,20^; however, the effect of oral cancer on neuronal TRPV1 expression and sensitization is unclear. We have previously demonstrated a sex difference in pain secondary to oral cancer; male mice demonstrate significantly less oral cancer-induced nociceptive behavior due to a neutrophil-mediated endogenous opioid mechanism^21,22^. Thus, due to the potential confounding effects of the immune system on oral chemosensitivity, male mice were analyzed separately for behavioral experiments. We used the oral cancer tongue xenograft model in tandem with the two-bottle choice assay to determine oral chemosensitivity during tumor growth. Naïve athymic nude mice were housed 3 per cage and baseline consumption (vehicle water) was measured for 7 days followed by HSC-3 inoculation or sham injection. On post inoculation (PID) 3, one vehicle water was replaced with 1 µM capsaicin water and consumption was measured by weight every 7 days for 4 weeks (Fig. 2A). We found an interaction between time and treatment in female mice only (F(3,12) = 10.22, p = 0.0013). Tumor-bearing female athymic nude mice demonstrated greater aversion to 1 µM capsaicin in the drinking water compared to sham at 3 (p = 0.0343) and 4 (p = 0.0001) weeks post inoculation (Fig. 2B). Total water consumption was similar over levels of time and treatment (Fig. 2C; F(3,12) = 1.389, p = 0.294). Consistent with our previous studies, we found no interaction between time and treatment in tumor-bearing male mice (Suppl Fig. 1, F(3,12) = 0.522, p = 0.675). In addition to oral sensitivity, we tested the hypothesis that capsaicin is driving pain in the oral cavity secondary to oral cancer by assaying CPA in mice that were conditioned with oral swabbing of vehicle (0.1% EtOH) followed by low dose 500 nM capsaicin (Fig. 2D). Pre-conditioning (baseline) times did not differ between the vehicle-paired chamber and the capsaicin-paired chamber (p = 0.39). Tumor-bearing female mice treated with oral capsaicin displayed CPA (Fig. 2E, F(1,8) = 14.13, p = 0.006); they spent less time in the capsaicin-paired chamber compared with either the pre-conditioning baseline (p = 0.019) or post-conditioning vehicle-paired chamber (p = 0.001; Fig. 2E). Oral capsaicin had no effect on sham female mice (Fig. 2F; F(1,8) = 3.02, p = 0.121) or in tumor-bearing male mice (Suppl Fig. 1, F(1,8) = 0.063, p = 0.808) or sham male mice (Suppl Fig. 1, F(1,8) = 1.187, p = 0.308).

### Oral cancer-induced increase in TRPV1 protein expression in tongue primary afferents

**Figure 3:**
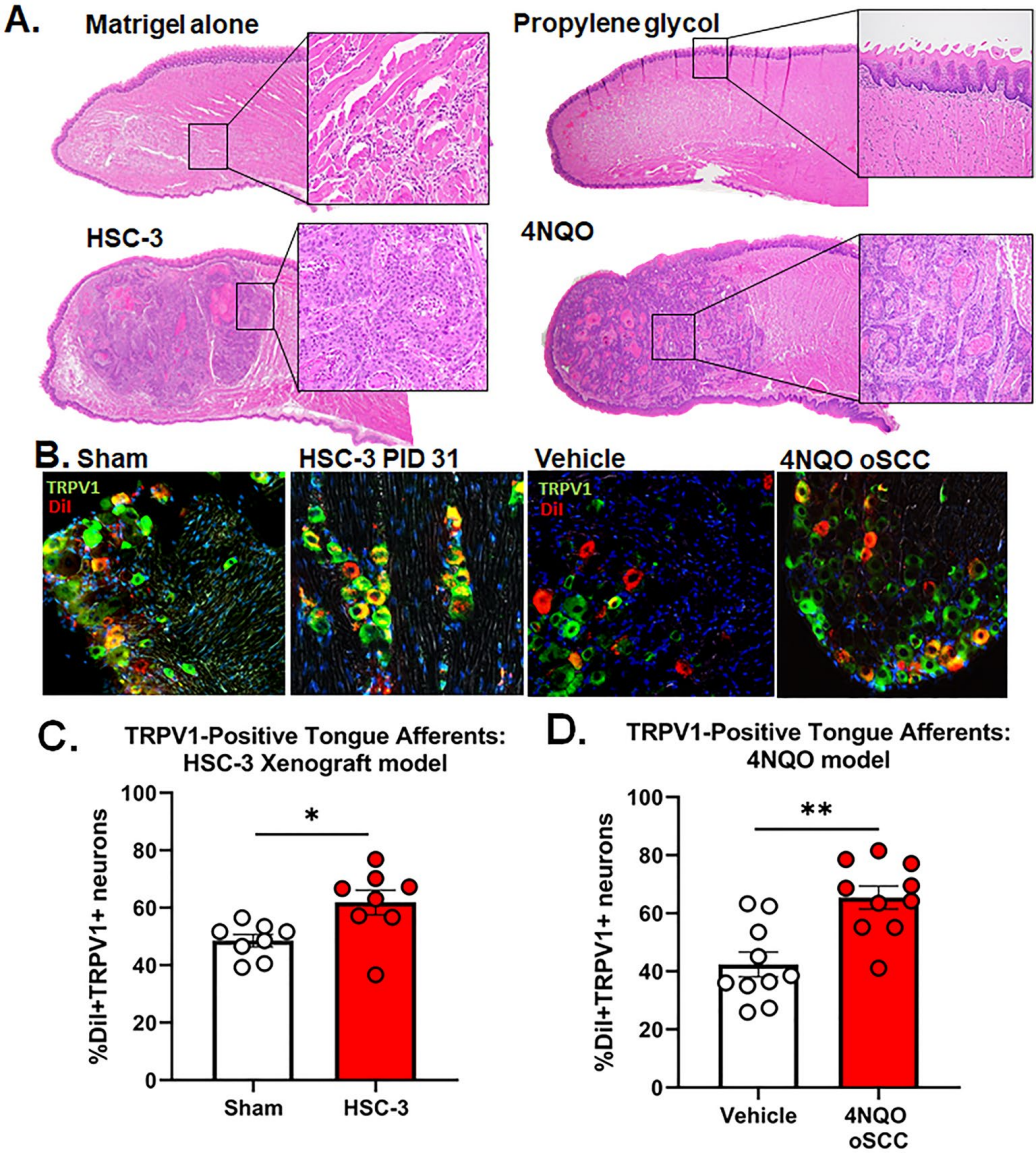
Oral cancer-induced increase in TRPV1 protein expression in tongue primary afferents. (**A**) Representative images of 5 µm tongue sections stained with hematoxylin and eosin from sham (*i.e.,* Matrigel alone) and HSC-3-tumor bearing mice at PID 31, as well as propylene glycol- and 4NQO-treated mice at week 28. (**B**) Representative images of TRPV1-like immunoreactivity in retrograde labeled (DiI) tongue primary afferent neurons from a section from female sham and HSC-3 xenograft at PID 31 as well as a vehicle treated and 4NQO-treated female mouse with oSCC, 10 × magnification. (**C**) Pooled data from male and female mice indicates an increased percentage of DiI + neurons co-labeled with TRPV1 (green) from HSC-3 xenograft (n = 4/sex) but not sham (n = 4/sex) mice at PID 31. *p < 0.05. (**D**) Pooled data from male and female mice indicates an increased percentage of DiI + neurons co-labeled with TRPV1 in 4NQO-induced oSCC (n = 4 female, 6 male) mice, but not vehicle treated mice (n = 4 female, 6 male).**p < 0.01.

Previous findings in a bone cancer mouse model found increased TRPV1 expression in dorsal root ganglia (DRG) neurons innervating the bone^20^. We hypothesized that the increase in capsaicin-induced aversion behavior was due to an increase in TRPV1 protein expression in tongue trigeminal afferents. We analyzed TRPV1 protein expression in the xenograft and 4NQO-induced oral cancer models, as well as the associated controls (sham or Matrigel only and propylene glycol, respectively). Representative hematoxylin and eosin stained images of the cancer models and control tongues are shown in Fig. 3A. We used retrograde labeling from the tongue using DiI and immunohistochemistry in TG sections from tumor-bearing mice (n = 4/sex) compared to sham control (n = 4/sex). There was no interaction between sex and treatment (F(1,12) = 1.744, p = 0.2112), therefore, sexes were pooled. DiI + TRPV1 + neurons increased only in tumor-bearing mice (Fig. 3B,C; t(14) = 2.187, p = 0.046). We have previously demonstrated that the immune system plays an important role in oral cancer pain^23^. To determine if the lack of an intact immune system in athymic nude mice contributes to cancer-induced changes in TRPV1 expression, we measured TRPV1 expression in wildtype mice with 4NQO-induced oral SCC (oSCC, n = 4 female, 6 male) and vehicle treated mice (n = 4 female, 6 male). There was no interaction between sex and treatment (F(1,16) = 0.918, p = 0.352), therefore, sexes were pooled. Similar to the xenograft model, DiI + TRPV1 + cells increased only in tumor-bearing mice treated with 4NQO; Fig. 3D, t(18) = 1.495, p = 0.0005).

### Oral cancer-induced increase in TRPV1 function

**Figure 4:**
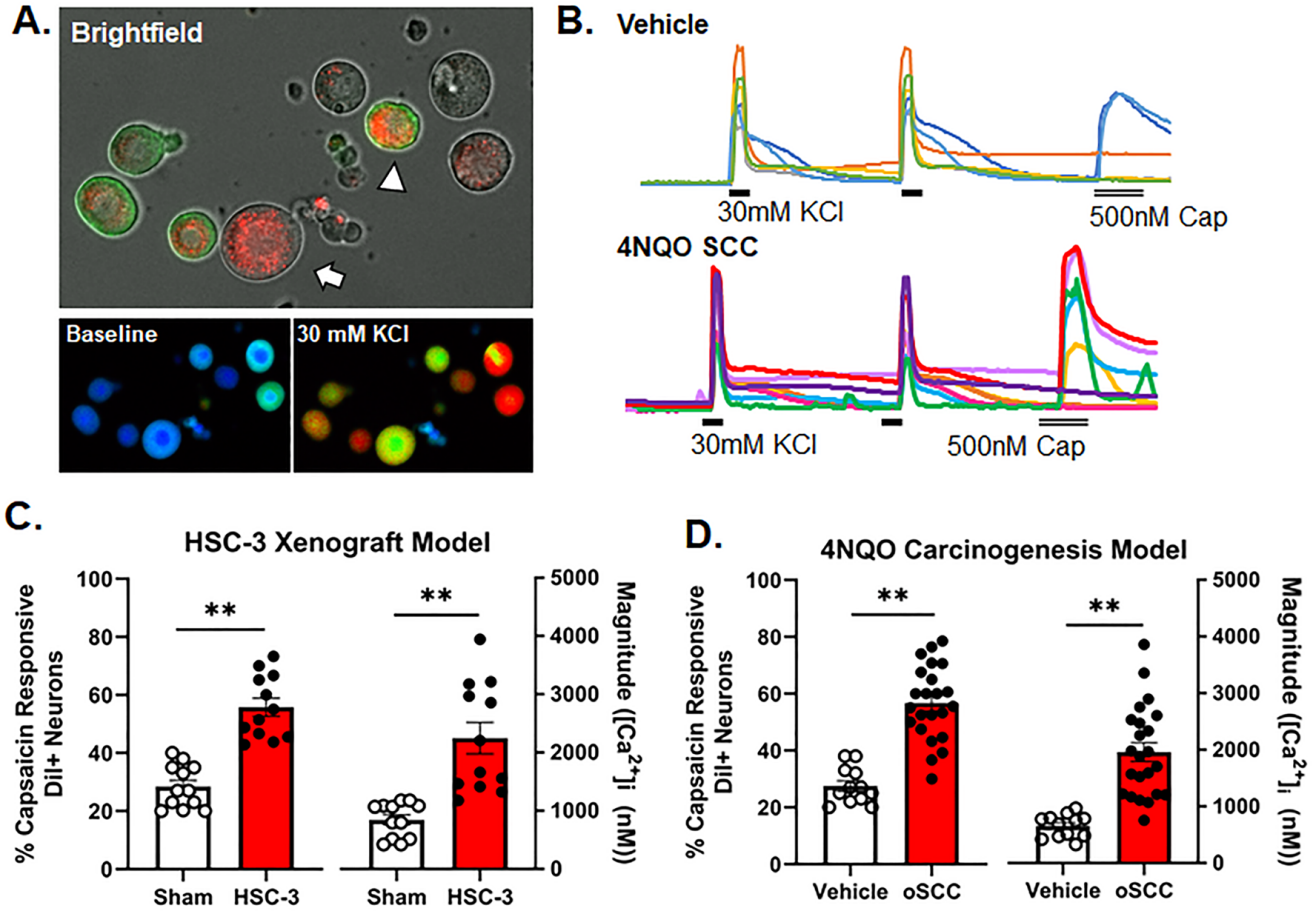
Oral cancer-induced increase in TRPV1 function (**A**) Image of dissociated TG neurons (bright field); a subset is retrograde labeled tongue afferents (DiI, white arrow) and IB4 nonpeptidergic (arrow head). (**B**) Traces of 30 mM KCl and 500 nM CAP-evoked transients from tumor bearing female mice. Ca^2+^ imaging was done at 20X magnification in fields containing ≥ 5 neurons and at least 2 coverslips were tested per mouse. For (**C**,**D**), which represent the HSC-3 xenograft and 4NQO models, respectively, each dot represents the average number of responders or the average Ca^2+^ transient magnitude of neurons tested from an individual mouse. The percentage of DiI + neurons that responded to CAP and the transient magnitude (peak-baseline) was increased in HSC-3 cancer-bearing mice (n = 12; 6/sex) compared to sham (n = 12; 6 mice/sex). **p < 0.01. Δ[Ca^2+^]_i_ = change in intracellular Ca^2+^ concentration. The percentage of DiI + neurons that responded to CAP and the transient magnitude (peak-baseline) in CAP-evoked transients was increased in mice with 4NQO induced oSCC (n = 22; 10 male, 12 female) compared to vehicle-treated mice (n = 12; 6/sex).**p < 0.01.

Next, we sought to determine cancer-induced changes in neuronal TRPV1 function. In dissociated retrograde labeled trigeminal neurons from mice, we quantified the percentage of retrograde labeled neurons that elicited a Ca^2+^ transient in response to 500 nM capsaicin as well as measured the magnitude of the capsaicin-evoked Ca^2+^ transient (Fig. 4A,B). We used neurons from mice with HSC-3 xenograft tumors (n = 6/sex) as well as 4NQO-treated mice with oSCC (n = 11 male,12 female). Sham athymic mice (n = 6/sex) and vehicle-treated C57BL/6 mice (n = 6/sex) respectively were used as controls. There was no interaction between sex and treatment for either model (xenograft: F(1,19) = 2.066, p = 0.167; 4NQO: F(1.31) = 1.400, p = 0.246), therefore, sexes were pooled. Oral cancer evoked an increase in TRPV1 function and magnitude of response compared to controls. The percent of DiI + neurons in which capsaicin evoked a Ca^2+^ transient > 20% of baseline was increased in neurons from mice with HSC-3 xenograft (t(21) = 6.829, p < 0.0001 Fig. 4C) as well as 4NQO-treated mice with oSCC (t(33) = 7.296,p < 0.0001, Fig. 4D). The magnitude of the capsaicin-evoked Ca^2+^ transient, defined as the peak Ca^2+^ response minus baseline Ca^2+^ concentration, increased in mice with HSC-3 xenograft (t(21) = 4.716, p = 0.0001 Fig. 4C), as well as 4NQO-treated mice with oSCC (t(33) = 5.508, p < 0.0001; Fig. 4D) but not in their respective controls.

### PAR_2_-induced sensitization of the capsaicin-evoked Ca2+ transient

**Figure 5:**
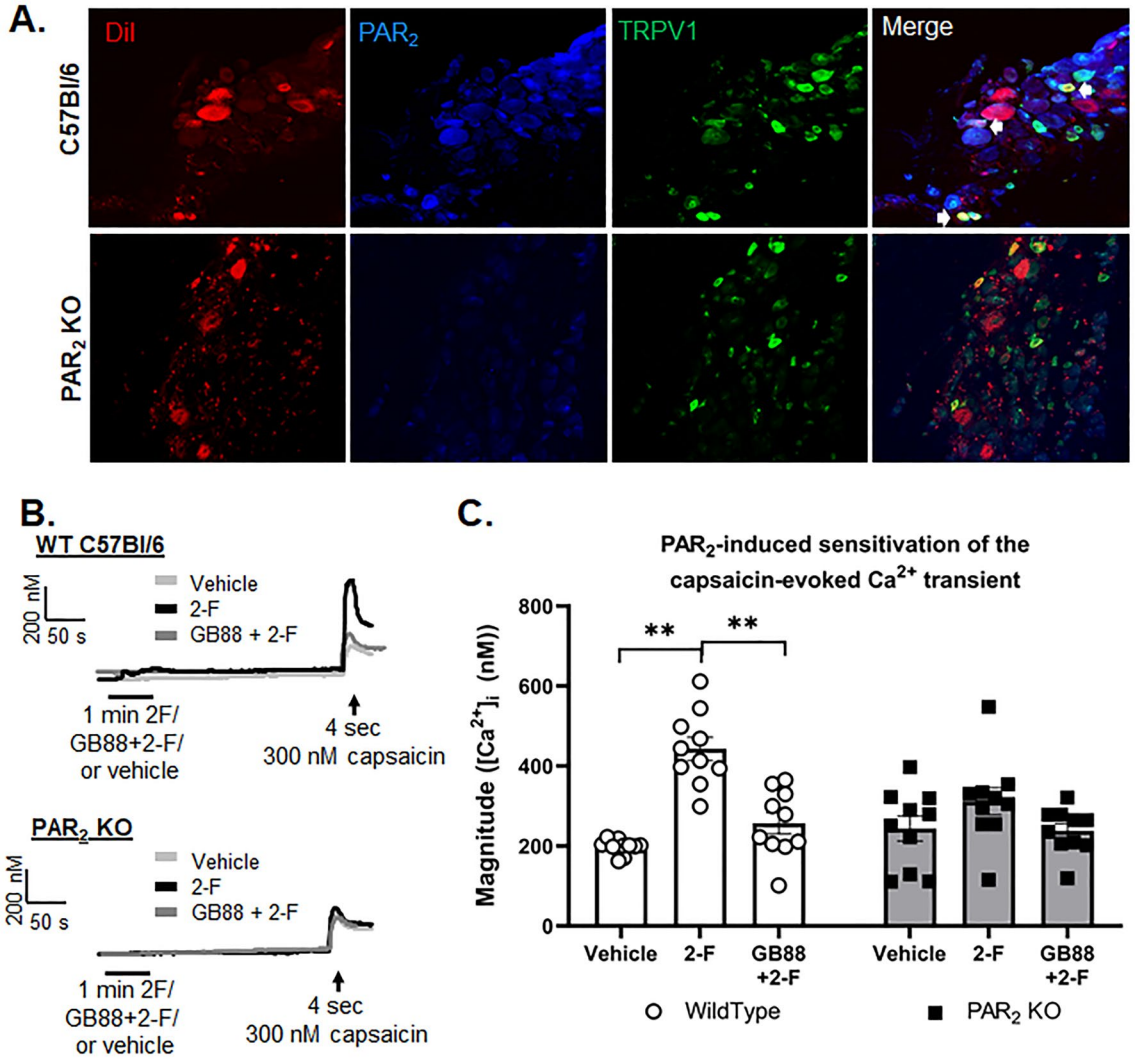
PAR_2_-induced sensitization of the capsaicin-evoked Ca^2+^ transient. (**A**) Triple labeling of PAR_2_ + TRPV1 + tongue primary afferent neurons using fast blue as retrograde tracer. (**B**) Representative traces from retrograde labeled tongue trigeminal neurons from (**C**) female wildtype C57BL/6 mouse or PAR_2_ global knockout mouse. TGs were dissociated and neurons were loaded with Fura-2AM for Ca^2+^ imaging. After 30 s of baseline recording, neurons were treated for 1 min with either vehicle (normal bath, light gray), PAR_2_ agonist, 2-F (100 nM, black) or pre-treatment with PAR_2_ antagonist, GB88 (10 μM), followed by 2-F (dark gray). The drug was washed out for 5 min and the neuron was challenged with capsaicin (300 nM, 4 s). (**C**) Treatment with 2-F resulted in increased capsaicin-evoked Ca^2+^ transient amplitude (peak–baseline) in tongue neurons from male and female wildtype mice (n = 10; 5/sex). There was no effect of 2-F treatment in tongue neurons from PAR_2_ KO mice (n = 10; 5/sex). **p < 0.01.

The tumor microenvironment is rich in protease and proteolytic peptides that directly activate PAR_2_ on sensory neurons. PAR_2_ activation has been shown to modulate TRPV1 activity^18,24^. To determine whether PAR_2_ and TRPV1 are co-expressed in tongue trigeminal neurons, we localized the proteins by immunofluorescence in tissue sections and measured the capsaicin-evoked Ca^2+^ transients in TG cultures using selective PAR_2_ pharmacology. Immunoreactive PAR_2_ and TRPV1 were colocalized in retrograde labeled tongue afferents in sections from TG from adult C57BL/6 mice and PAR_2_ KO mice (Fig. 5A); 33.8 ± 1.5% of retrograde labeled tongue afferents co-expressed PAR_2_ and TRPV1 protein (n = 3 mice). No PAR_2_ and TRPV1 immunoreactive overlap was identified in PAR_2_ KO mice (n = 3 mice). Next, we examined whether PAR_2_ activation sensitizes TRPV1-mediated Ca^2+^ transients in dissociated TG neurons. Capsaicin evoked a Ca^2+^ transient > 20% of baseline in 28.6 ± 2.5% of DiI + neurons responding to 30 mM KCl (n = 105 neurons total) in both wildtype and PAR_2_KO mice; there was no difference in the number of capsaicin-responsive neurons between genotype. The magnitude of the Ca^2+^ transient evoked by capsaicin varied with the interaction of drug treatment and genotype (F(2.54) = 5.952, p = 0.005). Pre-treatment with the specific PAR_2_ ligand, 2-F (100 nM), potentiated the response to capsaicin applied 5 min later by 123.52 ± 23.2% (n = 5/sex) in neurons from wild-type mice compared to neurons pre-treated with vehicle (p < 0.0001; Fig. 5B,C). Furthermore, pre-treatment with the PAR_2_ antagonist, GB88 (10 μM), inhibited the 2-F-induced potentiation of the capsaicin-evoked transient by 41.98 ± 17.7% only in neurons from wildtype mice (n = 5/sex; p < 0.0001). There was no effect of 2-F (p = 0.160) or GB88 (p = 0.119) on the capsaicin-evoked transient in PAR_2_KO mice (n = 5/group/sex, Fig. 5B,C).

### TRPV1-induced aversion behavior in the presence of oral cancer is mediated by PAR_2_

**Figure 6:**
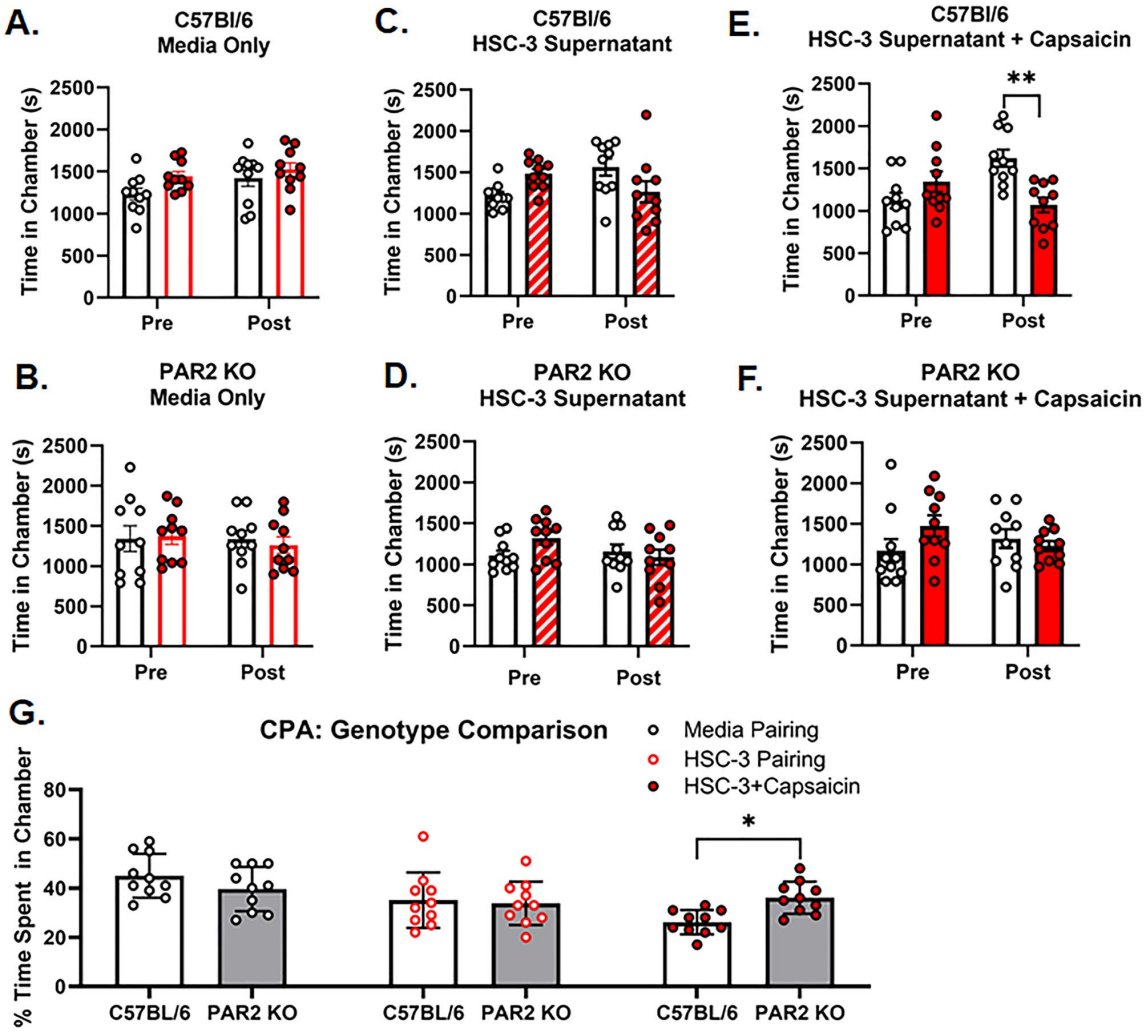
TRPV1-induced aversion behavior in the presence of oral cancer is mediated by PAR_2_. C57BL/6 or PAR_2_KO female mice were conditioned with either no injection or one of the following: saline injection, oral cancer supernatant injection into the tongue, or oral cancer supernatant followed by oral swabbing of 500 nM capsaicin 1 h post injection. The percent time spent in chamber was calculated as (time spent in the paired chamber / total time spent in both chambers post conditioning *100). (**A–F**) Conditioned place aversion was evident in C57BL/6 mice treated with HSC-3 supernatant + 500 nm capsaicin but not in other treatment types or PAR_2_KO mice. *p < 0.05, **p < 0.01. (**G**) Conditioned place aversion was evident only in C57BL/6 mice in the chamber paired with HSC-3 supernatant + 500 nM capsaicin (genotype by treatment interaction, p = 0.0170).

Lastly, we sought to determine if PAR_2_ mediates the TRPV1-evoked nociception secondary to oral cancer. We used CPA assay in tandem with the acute oral cancer pain model to test the hypothesis that PAR_2_ activation is required for capsaicin-induced aversion behavior in the presence of oral cancer. The acute supernatant model was used to quantify oral cancer-induced nociceptive behavior in the absence of tumor burden and illness associated with carcinogenesis. We first tested whether HSC-3 cell line supernatant alone evokes aversion behavior in wildtype C57BL/6 and PAR_2_KO female mice. Based on previous findings using hindpaw von Frey, HSC-3 supernatant-induced sensitization of primary afferents lasts for about 24 h^3^. While a “cell culture media only” injection group is the more appropriate for supernatant injection, multiple injections into the tongue per day over three days confounded the aversion behavior induced by the cancer supernatant. Therefore, for these CPA experiments, mice (n = 10/group) were conditioned with light anesthesia (2–2.5%) and no injection followed by either submucosal injection of cell line media or HSC-3 cell line supernatant (30 µl) into the tongue. Two-way ANOVA analyses showed that injection with cell line media does not evoke aversive behavior in either C57BL/6 (F(1,18) = 0.581, p = 0.456) or PAR_2_KO (F(1,18) = 0.175, p = 0.681) mice (Fig. 6A,B). On the contrary, conditioning with HSC-3 cell culture supernatant evokes mild aversion behavior in C57BL/6 mice (Fig. 6C; interaction: F(1,18) = 9.893, p = 0.006; Holm Sidak: p = 0.051), but not in PAR_2_KO mice (Fig. 6D; (F(1,18) = 2.103, p = 0.164). Oral cancer supernatant contains proteases which can activate neuronal PAR_2_^3,17^. We hypothesize that low dose capsaicin swabbing potentiates oral cancer-induced aversion behavior in a PAR_2_ dependent manner. To test this, mice were conditioned with no injection plus an oral swabbing of vehicle (0.01% EtOH) followed by HSC-3 cell line supernatant injection into the tongue plus, 1 h after injection, an oral swabbing of 500 nM capsaicin. Wildtype C57BL/6 mice demonstrated aversion to the chamber paired with HSC-3 supernatant plus capsaicin (Fig. 6E; interaction: F(1,18) = 10.78, p = 0.004; Holm Sidak: p = 0.001) whereas PAR_2_KO mice did not (Fig. 6F; (F(1,18) = 3.203, p = 0.090). Furthermore, the percent time spent in the treatment chamber varied with the interaction of treatment and genotype (Fig. 6G; F(2,54) = 4.401, p = 0.017); C57BL/6 mice spent less time than PAR_2_KO only in the HSC-3 supernatant plus capsaicin chamber (p = 0.033).

## Discussion

We report that oral cancer sensitizes TRPV1 through a PAR_2_-mediated mechanism. We propose that proteases cleave and activate PAR_2_ on primary afferent neurons in the oral cancer microenvironment, which sensitizes TRPV1 on the same neurons. Back labeled, anatomically relevant oral nociceptors respond to oral cancer by overexpressing TRPV1. We study expression of TRPV1 in two oral cancer mouse models. The xenograft model is created with a human oral cancer cell line which has implications for the study of human oral cancer. The carcinogen-induced oral cancer mouse model involves transformation of oral keratinocytes to malignant oral keratinocytes, similar to what would occur in oral cancer patients, and can be generated in mice with an intact immune system. Pharmacologic activation of PAR_2_ on oral nociceptors prior to the application of capsaicin enhances calcium influx. We employed two operant assays—drinking aversion and CPA—to confirm capsaicin sensitivity in oral cancer models; we used the former assay to confirm that PAR_2_ mediates oral cancer-induced capsaicin sensitivity.

Our results accord with those from two other studies that support the role of TRPV1 in oral cancer pain^19,25^. Ruparel and colleagues reported that three oral cancer cell lines, including HSC-3, a cell line we used in our study, secrete lipids that produce nocifensive behavior and thermal hypersensitivity through TRPV1^19^. The role of TRPV1 in bone cancer pain was demonstrated several years earlier when Mantyh and colleagues used pharmacologic antagonism and genetic disruption of TRPV1 to reverse spontaneous and functional pain in early and late stages of cancer progression^4^. Similar to our findings with tongue cancer and TG neurons, cancer-induced over expression of TRPV1 occurs in the DRG in a bone cancer pain model^20^. Cancer induces TRPV1-mediated functional changes as well. We demonstrated that back labeled neurons in two separate oral cancer models exhibit an elevated response to capsaicin. This effect is similar to the elevated response to capsaicin exhibited by neurons co-cultured with fibrosarcoma cells^26^.

An understanding of the mechanism, and the second messengers that mediate TRPV1 sensitization, following PAR_2_ activation might suggest a method for pharmacologic antagonism and treatment for oral cancer pain. While the mechanism of PAR_2_-mediated TRPV1 sensitization on tongue afferent neurons in the setting of oral cancer is not known, the mechanisms described for other conditions and neurons provide insight. PAR_2_ sensitization of TRPV1 occurs in TG, DRG and nodose/jugular complex sensory ganglia^18,27,28^. Sensitization of TRPV1 involves phosphorylation by PKC and PKA, which enhances ion channel activity^29,30^. PKC mediates neuronal processing following cleavage of PAR_2_^31^. PAR_2_ activates PKC by producing diacylglycerol through phospholipase C following recruitment and activation of Gq proteins. Oral cancers secrete proteases that activate PAR_2_, including legumain. In previous work we found that legumain cleaves PAR_2_ and activates PKA; this cascade can subsequently phosphorylate and sensitize TRPV1^17,32^. PAR_2_ also increases the open probability (*P*_O_) of the capsaicin-induced single channel in vagal pulmonary sensory neurons^28^. PAR_2_ activation increases the excitability of rat pulmonary capsaicin-induced chemoreflex in the lungs of rats^28^. Trypsin, which cleaves PAR_2_, enhances the capsaicin-induced pulmonary chemoreflex response and potentiates the whole-cell inward current induced by capsaicin in isolated nodose/jugular small-diameter neurons that innervate the lungs^33^. Similar to our approach, Gu et al., studied anatomically, back labeled relevant sensory neurons. The in vivo effects of PAR_2_ activation on the TRPV1-mediated tussive response have also been demonstrated in guinea pigs^24^.

There are other mechanisms, in addition to PAR_2_, by which oral cancer could sensitize TRPV1. Tumor necrosis factor alpha (TNFα), a pro-tumor inflammatory cytokine, is well known to potentiate TRPV1 activity leading to allodynia and thermal hyperalgesia in DRG^34^ and Ca^2+^ influx in TG^35^. We have recently demonstrated that TNFα is overexpressed in human oral cancer tissues^36^, secreted from human oral cancer cell lines and upregulated in cancer tongue tissues collected from mice treated with 4NQO^23^. Nerve growth factor (NGF) and adenosine triphosphate (ATP) are also secreted by oral cancer and can sensitize TRPV1^37,38^. TRPV1 can be sensitized through increased expression and/or receptor phosphorylation at key intracellular residues. NGF activates p38, which increases TRPV1 translation in the cell body and leads to subsequent anterograde transportation of the ion channel to nerve terminals in the periphery^39^. ATP sensitizes TRPV1 on TG neurons through phosphorylation of serine residues^27^. These TRPV1 sensitization mechanisms (PAR_2_, ATP, NGF) likely overlap as it has been shown that PAR_2_ activation by trypsin increases ATP secretion through TRPV1 sensitization^40^.

Limitations of our study include the control used for the CPA experiments, our use of global knockout mice, and single sex behavioral experimental design. Because generation of the HSC-3 xenograft cancer model requires a nude athymic mouse, and we used PAR_2_ knockout mice on a C57BL/6 background to study the role of PAR_2_, we used the HSC-3 supernatant model^3^. With regard to our CPA control, our previous work demonstrates that HSC-3 supernatant-induced sensitization of primary afferents lasts for about 24 h^3^. While a media only injection group is a more appropriate control for the CPA experiments, multiple injections per day over three days would likely confound the aversive behavior induced by the cancer supernatant. To address this injection-related concern, we demonstrated in a separate experiment that a saline injection does not induce aversive behavior. To obviate the potential confound of multiple injections, we employed CPA using anesthesia only condition pairing. From our demonstration that back labeled tongue afferent fibers co-express PAR_2_ and TRPV1, along with our demonstration that PAR_2_ activation enhances the calcium influx induced by TRPV1, we infer that PAR_2_ and TRPV1 on sensory neurons mediate the capsaicin sensitivity observed in our cancer model. However, there is a possibility that PAR_2_ expression on non-neuronal cells could mediate capsaicin sensitivity. We did not use a conditional PAR_2_ knockout selective for afferent fibers; for the current study, we used a global knockout mouse. Now that we have PAR_2_-Na_v_1.8 mice, we plan to use these mice in our future oral cancer and capsaicin sensitivity studies. Lastly, we used female mice only for the behavioral experiments to determine if TRPV1-induced aversion behavior in the presence of oral cancer is mediated by PAR_2_. Including the present study, we have now demonstrated a sex difference in pain and chemosensitivity secondary to oral cancer. We hypothesize that male mice demonstrate significantly less oral cancer-induced nociceptive behavior due to a neutrophil-mediated endogenous opioid mechanism^21,22^ and such, pre-treatment with naloxone in male mice may reveal the oral cancer-induced chemosensitivity during tumorigenesis.

In naïve mice, we found that 36% of lingual afferent fibers express TRPV1. Previous work by Elitt et al*.* and Wu et al*.* revealed TRPV1 expression in lingual TG neurons (25% and 17%, respectively)^41,42^. Capsaicin sensitivity might be greater in humans with oral cancer than in our mouse oral cancer model. A recent study revealed that TRPV1 expression is higher in human DRG than in mouse DRG; approximately 75% in the former and 32% in the latter^43^. Our study provides support for chemosensitivity testing in oral cancer patients to gain insight into neuronal plasticity in the context of oral cancer.

## Methods

### Animals

Male and female adult (6–20 weeks) athymic nude mice (NU/J, *Foxn1*^*nu*^, stock #002019, Jackson Labs, Bar Harbor, ME, USA), and C57BL/6 mice (stock #027, Charles River, Wilmington, MA, USA), TRPV1 KO mice (B6.129X1-Trpv1^tm1Jul^/J, stock #003770) and PAR_2_KO mice (B6.Cg-*F2rl1*^*tm1Mslb*^*/J*, stock #004993, Jackson Labs) were used. Mice were maintained on a 12:12 h light cycle and were housed in group cages in temperature-controlled rooms with access to food and water. Researchers were trained under the Animal Welfare Assurance Program. Experimental procedures were approved by the New York University Institutional Animal Care and Use Committee and were conducted in line with the National Institutes of Health guidelines for the use of laboratory animals in research.

### Cell culture

Human oral cancer cell line, HSC-3 (human tongue oral SCC cell line, cell number JCRB0623, from the Japanese Collection of Research Bioresources Cell Bank), was utilized to produce the human oral cancer xenograft model in immunocompromised nude mice. Cells were maintained and cultured as previously described^44^. HSC-3 cells were cultured in 10 cm^2^ cell culture dishes at 37 °C with 5% CO_2_ in Dulbecco’s modified Eagle’s medium (DMEM, Gibco, Waltham, MA, USA) supplemented with 10% fetal bovine serum and penicillin/streptomycin (50 U/mL). When cells reached 70% confluency, the culture medium was removed, and cells were washed with 5 mL PBS without Ca^2+^ and Mg^2+^. Cells were collected and resuspended in DMEM:matrigel (1:1; 1 × 10^5^ cells/20 µL HSC-3) for tongue inoculation; HSC-3 cells were collected from passage 7. Supernatant was collected as previously described for the conditioned place aversion (CPA) nociceptive behavior assay^23^. Briefly, cells were cultured to 70% confluency, washed twice with 5 mL PBS without Ca^2+^ and Mg^2+^ and media was replaced with 3 mL of serum and antibiotic free culture media (DMEM for HSC-3). After 48 h, supernatant was removed and spun at 300 × g for 4 min to remove debris and injected into the lateral anterior portion of the tongue within 20 min.

### Oral cancer xenograft model

A murine xenograft tongue cancer model was used to determine TRPV1 sensitization during oral carcinogenesis using the drinking choice behavior assay. Mice were inoculated with 1 × 10^5^ HSC-3 cells prepared in DMEM:Matrigel (1:1, 30 µL) into the anterior lateral portion of the tongue, as previously described^21^. HSC-3 is a human tongue squamous cell carcinoma; the use of this cell line and the study of the xenograft model has the advantage of increased translational relevance. Cancer growth and associated nociceptive behavior develops in approximately 10 days to 2 weeks following inoculation^21,25,37^. On post inoculation day (PID) 31, all mice were anesthetized with isoflurane followed by transcardial perfusion with cold Ca^2+^/Mg^2+^ free Hank balanced salt solution (HBSS, Invitrogen) for TG harvest; harvested TG neurons were placed on ice cold HBSS for dissociation and acute culture. To prepare tissues for immunohistochemistry, mice were anesthetized with isoflurane followed by transcardial perfusion with ice cold PBS followed by 4% paraformaldehyde (PFA). The experimenters conducting the behavioral tests were blinded to the treatment groups; the allocation keys were held by NNS.

### 4-nitroquinoline-1-Oxide (4NQO) oral carcinogenesis model

To determine changes in TRPV1 expression and function during carcinogenesis in an animal with an intact immune system, which is an advantage of the carcinogen-induced oral cancer model, mice were offered carcinogen 4-nitroquinoline-1-oxide (4NQO; 100 µg/mL; Sigma Aldrich, St. Louis, MO, USA) in the drinking water or the equivalent dilution of the vehicle, propylene glycol for 16 weeks, which is required to induce transformation of oral keratinocytes to malignant keratinocytes^23^. The mice were then monitored weekly under light anesthesia for body weight, tumor incidence, location, and size for an additional 12 weeks. If mice lost greater than 20% body weight prior to week 28 following the start of exposure to 4NQO, they were euthanized and TG and tongue tissue were collected. By week 28, all mice were euthanized regardless of clinical evaluation and health status. Euthanasia, perfusion and TG and tongue tissue preparation was performed as described for the oral cancer xenograft model. Tongue tissue was harvested by using bone scissors to bilaterally cut the jaw and spring-loaded scissors were used to free the tongue muscle from the floor of mouth. The tongue was dissected back to the oropharyngeal region, fixed in 10% formalin, and processed for paraffin embedding and slide preparation. Four 5 µm hematoxylin & eosin-stained tongue sections separated by 100 µm were evaluated for the presence of papillary and invasive SCC^45,46^. Histopathologic analysis was performed by an oral and maxillofacial pathologist blinded to group identity; allocation keys were held by NNS While calcium microfluorimetry and immunohistochemical evaluation was completed on all samples, only mice with histologically confirmed invasive lesions were included in subsequent data analyses.

### Drinking choice behavior assay

Mice were tested for TRPV1-mediated oral aversion to capsaicin using a paired-preference drinking aversion assay. A total of 36 athymic nude mice (18 males and 18 females) were used for these experiments. Mice were housed by sex in groups of three mice per cage. Cages were fitted with two water bottles, and positions of the bottles were swapped daily. Mice were acclimatized by ad libitum access to water with 0.1% ethanol vehicle in both bottles for one week. After acclimatization, mice were inoculated with HSC-3 cells freshly prepared in DMEM:Matrigel (1:1, 20 µL) or DMEM:Matrigel (1:1, 20µL) alone in the sham group. On PID 3, each cage was fitted with one bottle that contained vehicle (0.1% ethanol) and another bottle that contained 1 µM capsaicin (Sigma-Aldrich, St. Louis, MO). After seven days, the volume consumed from each bottle was measured by weight (1 mL water = 1 g). Fluid consumption was measured weekly for four weeks. An index measuring aversion to capsaicin was calculated individually for treatment group (sham/tumor-bearing) and sex as previously described^47^. Solutions and cages were replaced with fresh solutions following each measurement. Oral aversion to capsaicin was determined by two-way ANOVA (time by treatment).

### Conditioned place aversion assay

CPA occurs by pairing a painful experience with a distinct context resulting in subsequent avoidance of the same contextual cues^48,49^. We use the CPA assay to measure aversive behaviors in response to three treatment groups, (1) injection of supernatant from oral cancer cell line culture, (2) low-dose capsaicin (500 nM, oral swabbing), and (3) supernatant injection followed by oral capsaicin swabbing. We performed a single trial CPA protocol. The 3-chamber CPA apparatus consists of two conditioning chambers with distinct tactile, visual, and olfactory cues, connected by a smaller neutral chamber that was brightly lit. The visual cues were horizontal stripe and dot wallpaper. The tactile cues were smooth and rough flooring. The olfactory cues were strawberry and mint. White noise was played to provide background noise and block out any extraneous sounds. On the first day (preconditioning) of the experiment, mice were introduced to the neutral chamber and allowed to explore all three chambers for 1 h. Baseline time spent in the chambers was measured using ANY-maze tracking software (Braintree Scientific, Braintree, MA, USA). Exclusion criteria included mice spending < 20% or > 80% time in a chamber. Drug-chamber pairings for conditioning were counterbalanced across subjects^50^. On the second, third and fourth days, conditioning treatment varied based on experimental group. All mice underwent light anesthesia (2–2.5% isoflurane) prior to all treatments. For the supernatant-induce aversion, mice received anesthesia only followed by confinement into the appropriate pairing chamber for 1 h, following which they were returned to their home cage. Four hours later, mice received either submucosal injection of cell line media or HSC-3 cell line supernatant (30 µl) into the anterior lateral tongue followed by confinement into the opposite pairing chamber for 1 h. For the capsaicin-induced aversion, mice received an oral swabbing of vehicle (0.1% EtOH) followed by confinement into the appropriate pairing chamber for 1 h, following which they were returned to their home cage. Four hours later, mice received oral swabbing of 500 nM capsaicin followed by confinement into the opposite pairing chamber for 1 h. Low dose 500 nM capsaicin was chosen as it was found to be non-aversive in naïve athymic nude mice during the two-bottle choice assay (data not shown). For supernatant/capsaicin-induced aversion, mice received an oral swabbing of vehicle (0.01% EtOH) followed by confinement into the appropriate pairing chamber for 1 h, following which they were returned to their home cage. Three hours later, mice received submucosal injection of HSC-3 cell line supernatant (30 µl) into the anterior lateral tongue. One-hour later the mice received oral swabbing of 500 nM capsaicin followed by confinement into the opposite pairing chamber for 1 h. On the fifth day, mice were once again allowed to freely explore the apparatus for 1 h. Time spent in each chamber was recorded by ANY-maze. The experimenter conducting the behavioral tests (IMW) was blinded to the treatment groups; the allocation keys were held by NNS.

### Primary culture

Bilateral TGs were isolated by removing the skull cap and brain to expose the TGs readily visible in the base of the cranium. Using spring-loaded scissors the TGs were dissected and placed into cold HBSS and dissociated based on the previously described protocol^51^. Briefly, TG were minced and incubated in HBSS containing collagenase type II (Gibco) and dispase type II (Gibco) at 37 °C for 20 min and mechanically dissociated using a fire polished glass Pasteur pipette. A 12.5% by 28% percoll gradient was used to separate myelin and nerve debris from TG neurons. Cells were then plated in DMEM/F12 (Gibco) containing 5% fetal bovine serum and antibiotics (penicillin/streptomycin, 50 U/mL) on 5 mm coverslips. Coverslips were flooded 2 h later with Leibovitz L-15 media (Gibco) containing 10% fetal bovine serum, 5 mM HEPES and 5 mM glucose, and used at room temperature. Experiments were performed within 8 h of tissue harvest.

### Calcium imaging

TG neurons plated on 5 mm coverslips were incubated with 5 μM Ca^2+^ indicator fura-2 AM (Invitrogen, Carlsbad, CA, USA) at room temperature for 30 min in a physiological salt buffer (normal bath, 130 NaCl, 3 KCl, 2.5 CaCl_2_, 0.6 MgCl_2_, 10 HEPES, 10 Glucose, pH = 7.4, 325 mOsm). Coverslips were placed in a recording chamber and continuously infused with normal bath at room temperature. Imaging was done at 20X magnification in fields containing ≥ 5 neurons and at least 2 coverslips were tested per mouse. To measure the neuronal Ca^2+^ response to TRPV1 activation, capsaicin was applied for 4 s. To measure the neuronal Ca^2+^ response to TRPV1 activation following PAR_2_ agonist 2-Furoyl-LIGRLO-amide (2-F), or PAR_2_ antagonist GB88, 2-F or 2-F + GB88 was applied for 1 min followed by a 5 min wash with normal bath and then capsaicin was applied for 4 s. To test for cell viability, 30 mM KCl was applied for 4 s at the end of each experiment. Drugs and KCl were applied with a fast-step perfusion system (switching time < 20 ms; Warner Instrument Co, Model SF-77B). A change in intracellular Ca^2+^ concentration ([Ca^2+^]_i_) ≥ 30% of baseline was considered as a response to treatment. The magnitude of the response was calculated as (Peak [Ca^2+^]_i_) – (Baseline [Ca^2+^]_i_). To account for biological variance, percentage of DiI + neurons that responded to capsaicin and the average magnitude of response across DiI + capsaicin-responsive neurons were calculated and the data for each mouse was reported. Fluorescence data was acquired by a Nikon Eclipse Ti microscope at 340 nm and 380 nm excitation wavelengths and analyzed with the TI Element Software (Nikon, Melville, NY, USA). [Ca^2+^]_I_ was determined from fura-2AM ratio. Calibration of imaging software to convert Fura-2 ratio following in situ calibration experiments was performed as previously described^52,53^.

### Immunohistochemistry

To determine changes in protein expression in TG ganglia neurons innervating the tongue, retrograde tracers were injected into the tongue 10 days prior to inoculation or administration of 4NQO or vehicle. Retrograde tracer 1,19-dioctadecyl-3,3,39,39 tetramethylindocarbocyanine perchlorate (DiI; Invitrogen, Carlsbad, CA) was dissolved to 170 mg/mL in DMSO, diluted 1:10 in 0.9% sterile saline, and injected bilaterally using a 30 g needle for a total volume of 5-7µL per tongue of adult male and female mice to label tongue afferents. Retrograde tracer, Fast Blue (Polysciences, Inc, Warrington, PA, USA), was dissolved using Milli-Q water to a final concentration of 2.0% and injected bilaterally using a 30 g needle for a total volume of 5-7µL per tongue of adult male and female C57Bl/6 mice to label tongue afferents. At least 10 days following tongue tracer injection, animals were euthanized through an overdose of inhaled isoflurane and perfused transcardially with phosphate buffered saline (PBS) followed by 4% paraformaldehyde (Sigma Aldrich). TG were dissected, postfixed for 1 h in paraformaldehyde, and cryoprotected in 30% sucrose PBS) at 4 °C. TG were embedded in Tissue-Tek OCT compound (Sakura Finetek, Torrance, CA), sectioned (8 µm), and mounted on Superfrost Plus slides (Fisher Scientific Company, Pittsburgh, PA). Sections were blocked for 1 h in 3% goat serum and incubated in primary antibody in PBS containing 1% bovine serum albumin overnight at room temperature. The primary antibodies used were as follows: guinea pig anti-TRPV1 (1:500, Alomone AGP-118, lot#ANO125), rabbit anti-PAR2 (1:250, Thermo Scientific PA5-77685, lot#VJ3105322). Slides were extensively washed in PBS and incubated in goat anti–rabbit or guinea pig secondary antibodies conjugated to cyanine 2 or 3 (Jackson ImmunoResearch, West Grove, PA) in PBS at 1:250 for 2.5 h, extensively washed, and cover-slipped with FluoroGel II containing DAPI (Electron Microscopy Sciences). Sections were photographed using NIS Elements software and a Nikon Eclipse Ti microscope. For quantification of TRPV1-positive neurons across treatment groups as well as triple labeling of retrograde tracer (DiI), TRPV1-positive and PAR_2_-positive neurons, TG neurons with distinct nuclei within the V3 region were counted in every fifth section across three different sections per animal from 3 individual animals with a 20 × objective and identical laser gain settings.

### Statistical analysis

An independent samples t-test or analysis of variance (ANOVA) was employed to evaluate the difference between groups defined by genotype and treatment, and mixed models ANOVA analysis was used when repeated measures over time were also included in the analysis. To adjust for multiple comparisons, the post hoc Holm-Sidak test statistic was employed. Statistical significance was set at p < 0.05. All statistical analyses were performed using Prism (version 8) statistical software (GraphPad Software Inc., San Diego, CA, USA). Results were presented as mean and standard error of the mean in box/scatter configuration to show the biological variability. Our study is reported in accordance with ARRIVE guidelines (https://arriveguidelines.org).

## Supplementary Information

Supplementary Information 1.

Supplementary Information 2.
