# Supplementary figures and images for: Oral cancer induced TRPV1 sensitization is mediated by PAR2 signaling in primary afferent neurons innervating the cancer microenvironment
Source: Sci Rep. 2022 Mar 8;12:4121. doi: 10.1038/s41598-022-08005-6 (PMC8904826; doi:10.1038/s41598-022-08005-6)

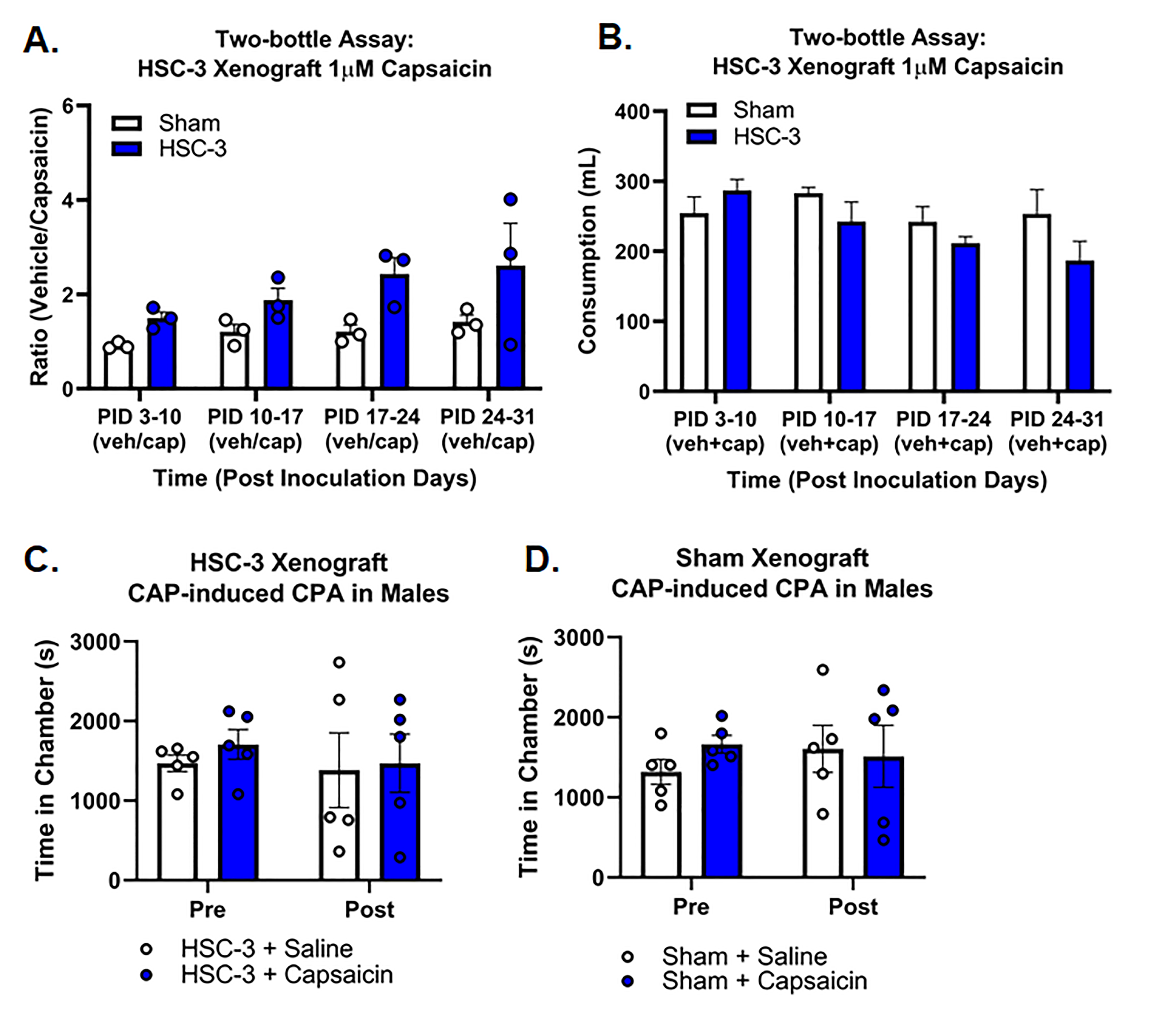

Supplement: Supplementary file 1 — Supplementary Information 1. [file 41598_2022_8005_MOESM1_ESM.tif]
