# Supplementary material for: Oral cancer induced TRPV1 sensitization is mediated by PAR2 signaling in primary afferent neurons innervating the cancer microenvironment
Source: Sci Rep. 2022 Mar 8;12:4121. doi: 10.1038/s41598-022-08005-6 (PMC8904826; doi:10.1038/s41598-022-08005-6)
Supplement: Supplementary file 2 — Supplementary Information 2. [file 41598_2022_8005_MOESM2_ESM.docx]

**Supplemental Figure 1.** **A)** Tumor-bearing male athymic nude mice demonstrate no greater aversion than sham animals to 1µM capsaicin in the drinking water at any time point post inoculation. Each dot represents a cage of three male mice. Two-way ANOVA, time by treatment interaction, p=0.675. **B)** There was no change in total water consumption with cancer progression in tumor-bearing mice, p=0.066. Baseline aversion behavior to either saline or 500nM capsaicin oral swabbing on post inoculation was assayed on days 22-24. On day 25, place aversion was defined as the difference in time spent in the CAP-paired chamber compared to saline-paired chamber. **C,D)** Tumor-bearing male mice (p=0.808) and sham-treated mice (p=0.310) spent similar amounts of time in the capsaicin-paired and vehicle-paired room, relative to baseline.
